# Effectiveness of a Conversational Chatbot (Dejal@bot) for the Adult Population to Quit Smoking: Pragmatic, Multicenter, Controlled, Randomized Clinical Trial in Primary Care

**DOI:** 10.2196/34273

**Published:** 2022-06-27

**Authors:** Eduardo Olano-Espinosa, Jose Francisco Avila-Tomas, Cesar Minue-Lorenzo, Blanca Matilla-Pardo, María Encarnación Serrano Serrano, F Javier Martinez-Suberviola, Mario Gil-Conesa, Isabel Del Cura-González, Dejal@ Group

**Affiliations:** 1 Healthcare Center Los Castillos Madrid Health Service Alcorcón Spain; 2 Healthcare Center Santa Isabel Madrid Health Service Leganes Spain; 3 Preventive Medicine and Public Health Department Rey Juan Carlos University Alcorcon Spain; 4 Health Care Center Perales del Rio Madrid Health Service Getafe Spain; 5 Health Care Center Panaderas Madrid Health Service Fuenlabrada Spain; 6 Healthcare Center Los Fresnos Madrid Health Service Torrejón de Ardoz Spain; 7 Healthcare Center Guayaba Madrid Health Service Madrid Spain; 8 Preventive Medicine Service Hospital Universitario Fundación Alcorcón Madrid Health Service Madrid Spain; 9 Research Unit Primary Care Assistance Management Madrid Health Service Madrid Spain; 10 Research Network on Health Services in Chronic Diseases Instituto de Salud Carlos III (ISCIII) Madrid Spain; 11 See Acknowledgments

**Keywords:** smoking, tobacco cessation, primary care, smartphone use, chatbot, dialog systems, artificial intelligence, tobacco, mHealth, primary care

## Abstract

**Background:**

Tobacco addiction is the leading cause of preventable morbidity and mortality worldwide, but only 1 in 20 cessation attempts is supervised by a health professional. The potential advantages of mobile health (mHealth) can circumvent this problem and facilitate tobacco cessation interventions for public health systems. Given its easy scalability to large populations and great potential, chatbots are a potentially useful complement to usual treatment.

**Objective:**

This study aims to assess the effectiveness of an evidence-based intervention to quit smoking via a chatbot in smartphones compared with usual clinical practice in primary care.

**Methods:**

This is a pragmatic, multicenter, controlled, and randomized clinical trial involving 34 primary health care centers within the Madrid Health Service (Spain). Smokers over the age of 18 years who attended on-site consultation and accepted help to quit tobacco were recruited by their doctor or nurse and randomly allocated to receive usual care (control group [CG]) or an evidence-based chatbot intervention (intervention group [IG]). The interventions in both arms were based on the 5A’s (ie, Ask, Advise, Assess, Assist, and Arrange) in the US Clinical Practice Guideline, which combines behavioral and pharmacological treatments and is structured in several follow-up appointments. The primary outcome was continuous abstinence from smoking that was biochemically validated after 6 months by the collaborators. The outcome analysis was blinded to allocation of patients, although participants were unblinded to group assignment. An intention-to-treat analysis, using the baseline-observation-carried-forward approach for missing data, and logistic regression models with robust estimators were employed for assessing the primary outcomes.

**Results:**

The trial was conducted between October 1, 2018, and March 31, 2019. The sample included 513 patients (242 in the IG and 271 in the CG), with an average age of 49.8 (SD 10.82) years and gender ratio of 59.3% (304/513) women and 40.7% (209/513) men. Of them, 232 patients (45.2%) completed the follow-up, 104/242 (42.9%) in the IG and 128/271 (47.2%) in the CG. In the intention-to-treat analysis, the biochemically validated abstinence rate at 6 months was higher in the IG (63/242, 26%) compared with that in the CG (51/271, 18.8%; odds ratio 1.52, 95% CI 1.00-2.31; P=.05). After adjusting for basal CO-oximetry and bupropion intake, no substantial changes were observed (odds ratio 1.52, 95% CI 0.99-2.33; P=.05; pseudo-R^2^=0.045). In the IG, 61.2% (148/242) of users accessed the chatbot, average chatbot-patient interaction time was 121 (95% CI 121.1-140.0) minutes, and average number of contacts was 45.56 (SD 36.32).

**Conclusions:**

A treatment including a chatbot for helping with tobacco cessation was more effective than usual clinical practice in primary care. However, this outcome was at the limit of statistical significance, and therefore these promising results must be interpreted with caution.

**Trial Registration:**

Clinicaltrials.gov NCT 03445507; https://tinyurl.com/mrnfcmtd

**International Registered Report Identifier (IRRID):**

RR2-10.1186/s12911-019-0972-z

## Introduction

Tobacco addiction is the leading cause of preventable morbidity and mortality in the world, directly causing 7 million deaths annually. Should this trend continue, this figure would rise to 8 million deaths by 2030, mostly in developing countries [1].

Population studies repeatedly conclude that the majority of smokers would like to quit and the percentage of them who try every year is high [1]. Most tobacco users stop smoking without help, although professional interventions increase the number of attempts and use of effective medication, resulting in a 2- to 3-fold success rate in the long term [2].

Different interventions by health professionals have proven to be effective and efficient, with the best outcomes observed when behavioral and pharmacological treatments are combined [2,3]. However, only 1 in 20 cessation attempts is supervised by a health professional [3]. Almost 84% of smokers who attended a primary care within the Madrid Health Service in 2008 had not received any advice to quit smoking over the 3 months prior to the consultation [4], which is similar to reports from other countries [5,6]. Factors accounting for these low intervention rates have been identified, among which are the training deficit of professionals, their perception that these interventions are not very effective, and their lack of time to implement them [7].

More intensive clinical interventions yield higher cessation rates in the long term; however, they are more expensive, require specifically trained professionals, and entail more health care time, which are inconvenient for both health care providers and users, who occasionally cannot afford them [8]. The potential advantages of mobile technologies for health (mHealth) [9]—effectiveness, accessibility, portability, privacy, customization, time-sensitive interventions, access to social support, superior adherence, and enormous scalability potential—can circumvent these problems and facilitate tobacco-cessation interventions for public health systems.

Globally, the number of smartphones used is increasing. There are an estimated 5200 million cell phone users and an estimated 8 billion cell phone lines worldwide, which exceed the world population (penetration rate of 102%) [10], and these numbers are expected to continue rising. Smartphones have become the most frequent and most accessible form of computer in most countries. This relevance of information and communication technologies (ICTs) has even increased in the context of the COVID-19 pandemic due to the imposed social distancing, and tobacco addiction was not oblivious to the new circumstances.

Using ICTs also entails risks: access to websites and apps offering incomplete information or nonevidence-based therapies that are difficult to identify and can cause undesirable effects [11]; incorrect records due to anonymity of patients (including the difficulty to reach the target population) [12]; lack of nonverbal communication; potential discrimination (eg, impaired vision, illiteracy, socioeconomic level, age); feeling of invasion of privacy or of being controlled for the user; issues with adherence to treatment and its detection; costs generated from mobile data use; and problems regarding data protection, privacy, and confidentiality. Online interventions should complement and not substitute presential interventions for now [13], so creating a theoretical frame for correctly implementing this novel type of interventions is essential to guarantee minimum quality and homogeneity standards.

Evidence regarding the effectiveness of interventions for quitting smoking with the aid of ICTs is recent. A review by Whittaker et al [14], which included 26 clinical trials and 33,849 participants, concluded that automatized interventions with SMS text messages were effective, whether as the solely delivered intervention (relative risk [RR] 1.54, 95% CI 1.19-2.00) or in combination with other interventions (RR 1.59, 95% CI 1.09-2.33). That review was the first to incorporate 5 evidence-based, quality studies comparing the effectiveness of an app for cessation with low-intensity interventions (whether using apps or not), although the effectiveness of apps for increasing the abstinence rates in the long term was not proven (RR 1.00, 95% CI 0.66-1.52). A more recent review including 4 trials using apps reported similar results (RR 0.871, 95% CI 0.543-1.397) [15].

Chatbots are potentially useful tools for interventions using ICTs: they are virtual assistants that respond to questions and requests by the patients, have the ability to learn, and communicate with the user via messaging apps. They differ from apps in their structure (they do not require installation in the smartphone, and therefore do not occupy space in the terminal; the interface is like a chatroom; and programming-related costs and time are lower), usage (they are bidirectional communication tools), interaction (they are not limited to a series of actions set by the programmer), privacy (they do not collect data from the phone), and most importantly, they are artificial intelligence (AI) and natural language processing tools (Multimedia Appendix 1).

At the time of this writing, several clinical trials are being conducted to assess the effectiveness of a chatbot for quitting smoking [16,17] by comparing different interventions employing ICTs. However, this work considered that clinical practice was the best comparator because it is the standard treatment in our setting and the chatbot aims to reproduce the ideal professional-patient personalized interaction using novel technological support.

Given its easy scalability to large populations, chatbots are a potentially useful complement to usual treatment, with the consequent savings, whether they are integrated into a global plan for aiding smokers to quit or used alone.

The aim of this study was to assess the effectiveness of a chatbot, with an evidence-based design and including elements of AI and natural language processing, for helping people stop smoking compared with clinical practice in primary care.

## Methods

### Trial Design

This is a pragmatic, multicenter, controlled, and randomized clinical trial. The study was conducted in 34 primary health care centers in the Community of Madrid region (Spain) and had a follow-up period of 6 months. The Madrid Health Service provided care for 6,772,465 citizens in 262 health care centers when the trial was conducted (2019).

The study followed the CONSORT (Consolidated Standards of Reporting Trials) guidelines [18] (Multimedia Appendix 2). The trial protocol was previously registered [19] and no changes were made to the methods, intervention, or comparator, except for an additional analysis by subgroups, which was not included in the initial study design.

### Participants

Family practitioners and nurses from the 262 health care centers in the Madrid Health Service were offered to participate. The 248 health workers who volunteered as collaborators were informed of the study objectives, design, and methods, and received training about the fieldwork, handling of the data collection, and good practice in clinical research. Among them, only 161 professionals recruited participants.

Patients included were smokers aged over 18 years who visited their doctor or nurse for consultation for any reason during the inclusion period. Patients included must have smoked at least one cigarette over the previous month, accept professional help for quitting in the following month, own a smartphone in which a messaging app (Telegram) could be installed, confirm their availability to be reached for 6 months following the intervention, and provide informed written consent. Criteria for exclusion were showing significant communication barriers and participation in another dishabituation program or clinical trial simultaneously. Computer or internet illiteracy of patients was not assessed.

### Recruitment

Each collaborator had the objective of recruiting a minimum of 3 patients by offering participation to all smokers attending their consultation for any reason, in consecutive order, between October 1, 2018, and March 31, 2019. After checking compliance with the inclusion criteria, the patients were informed about the characteristics of the trial, and invited to participate and read an informative document (Multimedia Appendix 3). Patients who accepted to participate provided informed consent (Multimedia Appendix 4). Relevant data on patients who declined enrollment were collected (age, gender, and reason for declining).

**Figure 1:**
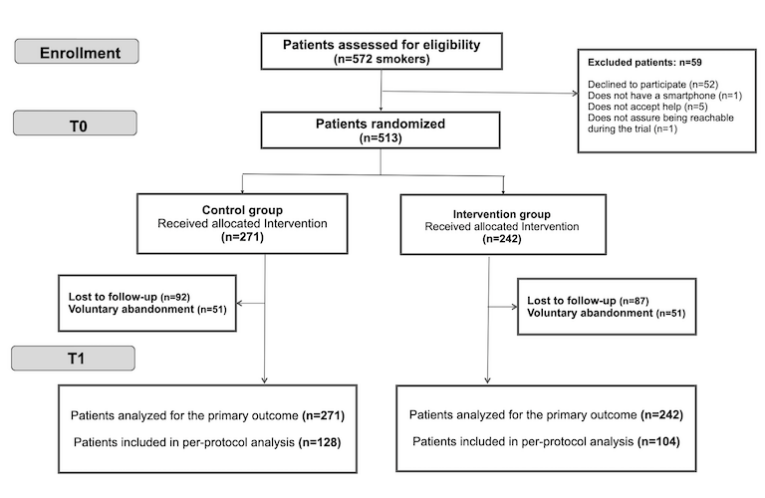
Study Flowchart.

Two visits were defined for patient data collection (Figure 1): baseline (T0) and at 6 months (T1). The health care collaborators collected participants’ data in a collection notebook designed ad hoc, which could be accessed from the work computer with a personal password. Additionally, professionals were responsible for the clinical follow-up of patients in the control group (CG) and keeping records of it at each visit.

### Randomization and Blinding

After providing informed consent and following the collection of baseline information, participants were randomly allocated to the intervention group (IG, chatbot) or CG (usual care) at the baseline visit (T0) via simple randomization software and without further restrictions. No other method was used to implement the random allocation sequence. The software generated a banner indicating the professional which group the patient had been assigned to, and a printable file with a password to access the chatbot for patients in the IG.

Given the nature of the intervention, patients and professionals were aware of their treatment allocation. All analyses were performed by trial statisticians and methodologists in the Madrid Primary Care Research Unit who were blinded to the group assignment.

### Intervention

The intervention strategy for both arms was based on the 5A’s (ie, Ask, Advise, Assess, Assist, and Arrange) in the US Clinical Practice Guideline [2]. During the recruitment phase, all patients who met the inclusion criteria were interviewed in person about their tobacco consumption and received advice to cease smoking from their doctor or nurse, who also inquired about their willingness to quit smoking. Those accepting to attempt cessation in the following month and agreed to participate in the trial were randomly assigned into the IG or the CG. Patients received a personal intervention that combined behavioral and pharmacological treatment and was structured in several follow-up visits, whether online via a chatbot or face-to-face with their assigned health care professional.

Patients in the CG received usual clinical practice that aided in their tobacco cessation process, which is based on scientific recommendations and protocols in the services portfolio of the Madrid Health System (Servicio 415, Atención al Consumo de Tabaco en el Adulto). The standard intervention comprised at least one appointment before the day of cessation and another visit after 1 month. Additional controls could be set, with frequency, intensity, and duration adjusted based on the professional criteria and patient needs.

Patients in the IG were offered an intervention with contents similar to the CG but delivered via a chatbot. A personal keyword allowed accessing the chatbot via Telegram, a widely used messaging app very similar to others, which makes it very easy to use and was chosen due to its better privacy warranties at the moment this trial was conducted. No instructions or recommendations were given to the chatbot users regarding timing, frequency, or intensity of use. No further appointments were set between the professional and the patient other than the follow-up at 6 months (T1), and no additional co-interventions were provided outside the trial setting.

Once the patients started the interaction upon their initiative, the chatbot guided them through the dishabituation process by establishing a 15-day period just before the cessation date with daily interactive contacts between the chatbot and patient. This was followed by encouraging and recognition messages by the chatbot after quitting that became more sporadic until completing the 6 months of abstinence. The contact frequency set by the chatbot varied depending on the time since quitting and patient characteristics (personal choice, type of tobacco use, personal risk situation, prescribed drug, abstinence-related symptoms, and evolution). The patient could contact the chatbot at any time and place, and decided the duration and frequency of interactions. There were no payments in any case or direction. The only expenditure for patients was that derived from consumed mobile data.

Dejal@bot was developed based on scientific evidence by doctors with expertise in tobacco use and ICTs between 2015 and 2018 (see Multimedia Appendix 5 for screenshots of the app). Its internal structure is a script recreating the interaction between a professional and a patient that takes numerous variants as required by the patient’s needs and characteristics. The chatbot is bidirectional and provides multimedia links to cessation advice (by providing access to evidence-based techniques with cognitive-behavioral, motivational, relapse-prevention, and problem-solving components); information about the prescribed medication for helping to quit; and advice on how to cope with abstinence-related problems and relaxation exercises in diverse formats, such as video, graphs, games, and web links (of note, all these are similar to the resources health care workers could offer to the patients in the CG). Dejal@bot also incorporates gamification elements (knowledge and skills acquisition) with a system for scoring points and obtaining badges that grant access to specific information depending on the abstinence period and personal needs. This feedback is complemented with messages of encouragement and emphasis on the achieved goals. The intervention was described in detail in the protocol [19] and in the TIDieR (Template for Intervention Description and Replication) checklist (Multimedia Appendix 6).

A pilot test was conducted prior to the beginning of the clinical trial to assess usability and to train the AI categories. The final version from the pilot study (February 2018) was implemented in the randomized controlled trial and its content was not modified at any stage.

No technical support service was available during the trial, which the authors believed would improve the chatbot accessibility and the retention rate.

Data collection was monitored weekly and the collaborators were contacted in case of incongruous or incomplete information.

The Dejal@bot structure is simple: (1) The user writes messages in the Telegram app installed into their smartphone; (2) Telegram anonymizes this message upon receipt by assigning an identification number to the user and forwards the message to the software installed in the research team server; (3) the software processes the message; (4) our reply is sent to Telegram; and (5) Telegram forwards the response to the user.

Telegram operates as a telephone service provider acting as a technological intermediate that does not store the content of the conversation. The chatbot only knows what the users say but there are no metadata in the conversation allowing their identification.

Dejal@bot works on an expert system that becomes more flexible in each decision by understanding the patients’ needs through a probabilistic interpretation of their message using techniques based on Bayes’ theorem. Therefore, the decision on which script to show next is not a prefixed sequence in a decision tree but rather works as follows: if the patient does not require special attention, the subsequent script is used in the order that has been preset in the expert system; however, if the patient requires special attention (eg, change of quit date, relapse, medication side effect), the Bayesian system detects this need and the specific script that has been preset for that particular case is used.

The AI layer has been generated using intelligent dictionaries of synonyms (48 classes with a total of 1127 terms), and therefore, the chatbot has learned different ways of expressing the same concept in natural language to respond appropriately regardless of the expression used by the user. The features and clinical content of the chatbot are presented in Textbox 1.

**Chatbot features and clinical content.**

**Features**

- Installed outside the smartphone (instead installed on the research team server).
- Communicate with the user via messaging apps.
- Easy to learn.
- Bidirectional communication.
- Respects the privacy of the user.
- Artificial intelligence.
- Natural language processing.
- Structured in several follow-up visits.
- Participants contact the chatbot at any time.
- Participants decide the duration and frequency of interactions.
- Multimedia links.
- Gamification elements.

**Clinical content**

- Evidence-based techniques with cognitive-behavioral, motivational, relapse-prevention, and problem-solving components.
- Variable contact frequency depending on time since quitting and patient characteristics.
- Information about the prescribed medication.
- Advice on how to cope with abstinence-related problems.
- Relaxation exercises.
- 15-day period just before the cessation date with daily interactive contacts.
- Encouragement and recognition messages by the chatbot after quitting with an emphasis on the achieved goals.

### Outcome Variables

The primary outcome was continuous abstinence at 6 months, which was biochemically validated by CO-oximetry, involving measurement of exhaled air in parts per million (ppm), following the recommendations in the Russell Standard [20]. Therefore, the patient must declare not having smoked in the previous 6 months and have a negative CO-oximetry result (<10 ppm) to be considered a “nonsmoker.”

The secondary outcomes were changes in quality of life, number of contacts between the therapist or the chatbot and the patient, and total time of interaction. The cost-utility assessment will be the subject of a separate analysis and paper. Adherence to pharmacological treatment could not be measured due to the low number of visits in the CG. No modifications to the trial outcome measurements were made after the trial commenced.

The EQ-5D-5L was used to assess quality of life. This validated generic instrument measures health-related quality of life on a visual analog scale (VAS) ranging from 0 to 100 (with higher ratings indicating higher quality of life) and includes 5 dimensions to assess mobility, self-care, usual activities, pain/discomfort, and anxiety/depression. Health condition is converted into a weighted Health Status Index, where *full health* receives a value of 1 and 0 stands for *death*. We collected data on the 5 dimensions and the Health Status Index proposed for our country [21].

Patient data were collected during the consultations with family practitioners and nurses at baseline (T0) and after 6 months (T1). At baseline, collaborators recorded sociodemographic variables (age, gender, economical level, educational level, and nationality); tobacco use (daily cigarette consumption, number of previous attempts to quit, CO-oximetry result in parts per million, cessation date, and level of nicotine dependence); and related variables (concomitant use of cannabis, prescribed pharmacological treatment, and type of pharmacological treatment indicated for the dishabituation process). Given the pragmatic nature of this trial, patients could contact the professionals or the chatbot at any time, and professionals could schedule follow-up visits with patients in the CG depending on both the recommendations in the portfolio of provided services and patient needs. Information regarding contact time and number of interactions was automatically recorded in the data collection notebook (CG) or by the chatbot (IG). No qualitative feedback was obtained from participants at any moment.

If the patient did not attend the 6-month follow-up visit (T1), their assigned professional tried to contact them on the phone up to 3 times to set an appointment, after which they were considered lost to follow-up.

No changes to trial outcomes were made after the trial commenced.

### Sample Size

The sample size was calculated based on the outcomes of the FTFT-AP trial [22], a recent clinical trial that assessed the effectiveness of usual clinical practice in our health care system and reported a continuous abstinence rate of 9.6% in the CG at 6 months. Considering a 2-fold success rate compared with the later study [14], an α error of 5%, and a power of 80%, the calculated sample size was 418 patients. With an estimated dropout rate of 10%, the final size was 460 smokers (230 in each arm).

### Statistical Analysis

Intention-to-treat analyses were performed by coding all losses to follow-up as smokers, as specified in the previously published protocol [19]. Stata 14 was used for the analyses.

Regression models (logistic or linear, as appropriate) were employed for analyzing the effect of the intervention on all outcomes and adjusted for robust estimators to account for patients recruited by clusters. Missing data were analyzed using the baseline-observation-carried-forward (BOCF) approach. The effectiveness of the intervention on the primary outcome was assessed via intergroup differences in the biochemically validated abstinence at T1 and T0, reported in proportions with the corresponding 95% CI. A sensitivity analysis was performed to compare the intention-to-treat and per-protocol analyses. Factors associated with confirmed continuous abstinence at 6 months were evaluated using a logistic regression model. Intragroup differences in quality of life between T1 and T0, as measured via the EQ-5D-5L VAS, were calculated as part of the analysis of secondary outcomes. Additionally, variables measuring the intensity of use were evaluated via intergroup differences in the average number of contacts and total interaction time. An analysis by subgroups was also conducted to account for the intensity of use with the chatbot or the contact intensity between patients and professionals.

Statistical tests for independent samples (Student *t* test and chi-square test) were applied for intergroup comparisons at baseline, and a repeated-measures ANOVA for related samples was used for evaluating intragroup differences and changes over time.

### Ethics Approval

This clinical trial was approved by the Ethics Committees for Clinical Research of the Community of Madrid (December 13, 2017; approval number: 23/17) and the University Hospital 12 de Octubre (Madrid, January 30, 2018; approval number: 18/054).

## Results

### Characteristics of Patients

Participants were recruited between October 1, 2018, and March 31, 2019. The last follow-up visit took place on October 31, of 2019. No critical “secular events” fell into the study period. The trial ended as planned in the protocol.

A total of 161 professionals collaborated in the trial and each recruited a mean of 3.18 (SD 1.69) patients. Of the 572 potentially eligible patients who had been invited to participate, 513 accepted, provided informed consent, and were thus enrolled in the trial. Participating patients showed characteristics similar to nonparticipants in terms of gender and age.

No significant differences were found between the IG and CG at baseline in terms of sociodemographic variables or those related to their tobacco consumption (Table 1). The average age was 49.8 (SD 10.82) years, 59.3% (304/513) were women, 93.8% (481/513) were Spanish, 68.2% (350/513) had completed secondary or university education, and 51.5% (264/513) earned under €17,000/year or US $18,100/year (nearly twice the minimum wage).

Concerning variables related to tobacco use, 10.1% (52/513) of patients reported moderate or high dependence on nicotine with Heavy Smoking Index values of 4-6 points and average consumption of 16.5 cigarettes/day (SD 7.75). Additionally, 3.3% (17/513) of patients were frequent cannabis users. The mean baseline CO-oximetry level was 15.11 ppm (SD 14.12) and mean attempts to quit were 2.48 (SD 2.91). Pharmacological treatment was prescribed for 49.3% (253/513) of patients. The mean baseline score on the EQ-5D-5L VAS was slightly higher in the CG (71.8, SD 18.1), compared with that in the IG (69.4, SD 18.5; P=.07), without intergroup differences in the questionnaire dimensions expressed by their relevant weighted Health Status Index [21].

Measurements were obtained at the follow-up visit (T1) for 232 (45.2%) patients, 42.9% (104/242) and 47.2% (128/271) in the IG and CG, respectively, without significant intergroup differences (Figure 1). The analysis of dropouts also did not show significant intergroup differences (Table 2).

**Table 1:** Clinical characteristics of patients.

Variable				Control group (n=271)			Intervention group (n=242)			P value	
Age (years), mean (SD)				50.66 (10.42)			49.01 (11.22)			.09	
**Gender, n (%)**										.64	
	Women		158 (58.3)			146 (60.3)					
	Men		113 (41.7)			96 (39.7)					
**Educational level, n (%)**										.67	
	Primary school or inferior		89 (32.8)			74 (30.6)					
	High school		132 (48.7)			116 (47.9)					
	University		50 (18.5)			52 (21.5)					
**Personal gross income (€^a^/year), n (%)**										.98	
	<8500		48 (17.7)			44 (18.2)					
	8500-16,999		94 (34.7)			78 (32.2)					
	17,000-25,499		73 (26.9)			66 (27.3)					
	25,500-33,999		36 (13.3)			34 (14.0)					
	>34,000		20 (7.4)			20 (8.3)					
**Country, n (%)**										.26	
	Spain		251 (92.6)			230 (95.0)					
	Other countries		20 (7.4)			12 (5.0)					
Number of daily cigarettes, mean (SD)				16.32 (8.04)			16.70 (7.43)			.59	
Previous tobacco withdrawal attempts, mean (SD)				2.37 (2.83)			2.60 (3.00)			.38	
Heavy Smoking Index, mean (SD)				2.65 (1.68)			2.71 (1.59)			.67	
**Cannabis use, n (%)**										.14	
	No		265 (97.8)			231 (95.5)					
	Yes		6 (2.2)			11 (4.5)					
**Pharmacological treatment^b^, n (%)**				130 (48)			119 (49.2)			.63	
	Simple nicotine replacement treatment		10 (7.7)			13 (10.9)					
	Combined nicotine replacement treatment		4 (3.1)			5 (4.2)					
	Bupropion		24 (18.5)			21 (17.6)					
	Varenicline		89 (68.5)			74 (62.2)					
	Others		3 (2.3)			6 (5.0)					
	No pharmacological treatment	141 (52.0)			123 (50.8)						
Baseline CO-oximetry (ppt), mean (SD)				15.51 (15.33)			14.70 (12.67)			.57	
EuroQol 5D-5L VAS^c^, mean (SD)				71.8 (18.1)			69.4 (18.5)			.14	
EuroQol 5D-5L index, mean (SD)				0.9 (0.2)			0.9 (0.2)			.11	

^a^€1=US $1.06.

^b^n=130 and 119, respectively, for the IG and CG.

^c^VAS: visual analog scale.

### Primary Outcome

Table 2 presents detailed intervention outcomes, from both the intention-to-treat (n=513) and per-protocol (n=232) analyses. In the intention-to-treat analysis using the BOCF at T1, an intergroup difference in the primary outcome was found, with a biochemically validated abstinence rate of 26.0% (63/242) in the IG versus 18.8% (51/271) in the CG (odds ratio [OR] 1.50, 95% CI 1.00-2.31; P=.05). After adjusting by CO-oximetry and bupropion intake, no substantial changes were observed (OR 1.52, 95% CI 0.99-2.33; P=.053; pseudo-*R*^2^=0.045).

In the explanative model, the factors found to correlate with the abstinence rate at 6 months were having received the chatbot intervention (OR 1.52, 95% CI 0.99-2.33; P=.053) and bupropion prescription (OR 2.81, 95% CI 1.49-5.32; P=.001). Baseline CO-oximetry level was not found to correlate with the abstinence rate at this time point (OR 0.96, 95% CI 0.94-0.99; P=.002; pseudo-*R*^2^=0.045).

**Table 2:** Abstinence rate at 6 months.

Groups and rate difference	CO-validated continuous abstinence (intention to treat)	CO-validated continuous abstinence (per protocol)
Control group, n/N (%)	51/271 (18.8)	51/128 (39.8)
Intervention group, n/N (%)	63/242 (26.0)	63/104 (60.6)
Rate difference (95% CI)	–7.2 (–14.4 to 0.0)	–20.7 (–33.4 to –8.1)
Odds ratio raw (95% CI)	1.5 (1.0 to 2.3)	2.3 (1.4 to 3.9)
P value	.05	<.001
Odds ratio adjusted (95% CI)^a^	1.52 (0.99 to 2.33)	2.35 (1.37 to 4.05)
P value	.05	.002

^a^Adjusted by baseline CO-oximetry and bupropion intake.

### Secondary Outcomes

In terms of quality of life, no intergroup differences were found at baseline on the VAS (71.8 in the CG versus 69.4 in the IG; P=.07). At 6 months, a significant difference on the EQ-5D-5L VAS was observed between those who had quit and those who had not (73.2 versus 64.7 points, respectively; P=.01) and also between patients in the IG and the CG (71.6 versus 66.7 points, respectively; P=.09), although statistical significance was not reached (P<.05).

In terms of variables related to intervention intensity, the mean total interaction time with the patients was 21.2 minutes (SD 18.3; 95% CI 19.0-23.4) in the CG and 121 minutes (SD 157.5; 95% CI 121.1-140.0) in the IG (P<.001), and the mean number of contacts was 2.92 (SD 1.89) in the CG and 45.56 (SD 36.32) in the IG (P<.001). Therefore, the mean interaction duration between the chatbot and patient was 2.65 minutes versus 7.26 minutes between the professional and patient. Contact was defined as the time attending consultation for cessation in the CG or as the chatbot-patient interaction plus the time for performing an activity in the IG, with a pause of more than 90 minutes being considered as the end of a contact.

Patients in the IG who had successfully quit interacted an average time of 176.1 minutes (CI 95% 124.4-227.7) versus 116.6 minutes (95% CI 65.6-167.7) for those who had not (P=.06). In the CG, the mean interaction time was 24.1 minutes (95% CI 19.1-29.2) for patients who had quit smoking versus 23.5 minutes (95% CI 19.8-27.3) for those who had not (P=.84). The average number of contacts in the IG was greater for patients who stopped smoking versus those who did not succeed (59.4 vs 40.9, respectively; P=.004), which was in contrast to the number of contacts in the CG (4.1 versus 3.6, respectively; P=.06).

An additional exploratory analysis by subgroups, which the protocol did not contemplate, was performed to assess the intensive use of the chatbot, defined as more than 4 contacts with the chatbot and over 30 minutes of total interaction time throughout the 6 months. The biochemically validated abstinence rate in the IG at T1 was significantly higher for patients who contacted the chatbot intensively versus those who did not (68.6% versus 40.9%, respectively; P=.02), which was in contrast to that observed in the CG (47.6% for patients having intensive contact with the health care worker versus 35.4% who were not; P=.30), for which also intensive contact was defined as more than 4 contacts and over 30 minutes of total interaction time throughout the 6 months.

Approximately half of the patients (130/271, 47.9% and 119/242, 49.2% in the CG and IG, respectively) received pharmacological treatment to quit smoking, with no observed intergroup differences. In the multivariate analysis, a relationship was found only between bupropion intake and biochemically confirmed abstinence at 6 months (OR 3.46, 95% CI 1.12-10.51).

## Discussion

### Principal Findings

Although no significant difference in smoking cessation rates was obtained, our results suggest an effect that is certainly promising (OR 1.5), with a difference in effect ranging from no effect (OR 1) or a 1% decrease (OR 0.99) in the raw result up to over 2-fold increase (OR 2.33). However, all values within the interval limits are reasonably compatible with the data, given the statistical assumptions made to calculate the interval. Therefore, these results must be interpreted with caution.

In terms of secondary variables, quality of life further improved for patients assigned to the chatbot intervention versus the CG, especially for those who succeeded in quitting. This finding is consistent with the higher abstinence rates observed in the IG and can be related to the success in quitting smoking rather than the assigned intervention. Nevertheless, the change observed in the IG, which showed a slightly lower quality of life at baseline, could be partly due to the intervention.

Both the total interaction time and the number of contacts were much greater in the IG than in the CG, although the average contact duration was shorter in the former group. The number of sessions and invested time are key factors for the effectiveness of interventions in smokers [2]. One premise for the chatbot intervention was that its automated use would facilitate an intensive intervention of characteristics similar to face-to-face interventions but without requiring as many resources. However, the setting of a chatbot-patient interaction is very different from a visit to the doctor or nurse in terms of type of interaction, duration (the chatbot is accessed easily and the intervention can be fragmented according to the patient needs), and activities performed by the patient (patients in the IG practiced behavioral techniques during the intervention time, whereas those in the CG did it at home and the invested time was not registered). This could partly justify the paradox that patients in the IG who did not succeed in quitting smoking spent more time contacting the chatbot than those who did quit in the CG, although further trials are required to clarify this aspect.

Patients in the IG who made intensive use of the chatbot (longer total interaction time and greater number of contacts) achieved significantly higher abstinence rates than those who did not, contrary to the CG, where no significant differences in abstinence rates were found between those having and not having intensive interaction. It appears that the majority of professionals conducted very homogeneous interventions in the CG, probably limited by their workload. However, the number of patients in the CG achieving continuous abstinence at 6 months was higher when the interventions reached intensive use (47 versus 35, respectively), despite not reaching clinical significance, probably due to the limited sample size.

The use of pharmacological treatment for tobacco cessation in usual practice yields over a 2-fold success rate for the same intervention duration [2]. In this trial, first-choice drugs were equally prescribed in both arms and the performed analysis showed that their effect was not considerable. In any event, the chatbot was designed to increase adherence to medication by providing accessible and tailored information, although this could not be properly measured due to the low number of follow-up visits in the CG.

The per-protocol analysis revealed a difference compared with the intention-to-treat analysis (Table 2), supporting the effectiveness of Dejal@bot, yet raising concerns about adherence to the chatbot, an aspect that must be improved.

In summary, accessibility, simplicity, ubiquity, and immediacy were components that probably favored longer interaction time between the chatbot and patients and a higher number of contacts, which are key factors for predicting long-term abstinence in interventions in smokers [2,3]. These, in addition to following usual practice guidelines, were the factors underlying the effectiveness of Dejal@bot.

Further trials are required to determine the components that mainly impact the effectiveness of the chatbot and which type of patients are susceptible to benefit from this type of intervention. Besides, more studies are needed with direct technical assistance for improving accessibility, as well as interventions for improving digital competencies in certain population groups, which would likely improve retention.

### Comparison With Other Studies

We identified only 2 clinical trials [16,23] that used chatbots to help people quit smoking. One study [16], without published outcomes at the time of this writing, aims at comparing a CG using SMS text messages with an IG using a chatbot (QuitBot) in smartphones for helping with the cessation process. Abstinence will be checked at 3, 6, and 12 months after the intervention via biochemical validation. The other study [23] was an experimental trial that added a chatbot to the already existing “Smoke Free App.” The trial compared the interaction between the user and the app with and without the chatbot. The inclusion of the chatbot in the app increased the self-reported abstinence at 1 month (OR 1.36, 95% CI 1.16-1.61; P<.001). Therefore, Dejal@bot is the first published clinical trial about the effectiveness of a chatbot for helping smokers to quit with biochemically validated abstinence outcomes in the long term. Multimedia Appendix 7 presents a list of articles on the use of apps and chatbots in the tobacco cessation process.

Given the absence of further similar studies, the outcomes of this trial were compared against those in several clinical trials using apps for helping to quit smoking. One study compared the effectiveness of 2 apps [24], one of them following the US clinical practice guideline [2] that achieved a 21.1% abstinence rate at 12 months versus an Acceptance and Commitment Therapy app that achieved a higher abstinence rate of 28.2% (OR 1.49, 95% CI 1.22-1.83; P<.001). These were self-reported and 1-time outcomes, unlike those in our trial.

In the study by Pallejà-Millán et al [25], participants in the IG who used the app regularly and correctly had a higher probability of being abstinent at 12 months (OR 7.20, 95% CI 2.14-24.20; P=.001) than those in the CG. That is the only trial comparing the use of an mHealth intervention with usual practice but, unlike ours, their design was based on conglomerates (health care centers) and not pragmatic. The obtained abstinence outcomes were statistically significant when contrasting correct versus incorrect use of the app, but not in the intergroup comparison. Of note, 34.2% (97/284) of patients in the IG did not enter the app for smoking cessation.

BinDhim et al [26] compared an interactive app (including a variety of options for cessation, evaluation of risks and benefits from quitting, motivational messaging, and diary of the cessation process) with a merely informative app. The abstinence results at 6 months were better with the intervention app (10.2% vs 4.8%; RR 2.02, 95% CI 1.08-3.81), although abstinence was self-reported.

Baskerville et al [27] compared an evidence-informed app for smoking cessation with an evidence-informed self-help guide for reducing the smoking prevalence among young adult smokers, and observed no differences at 6 months (OR 0.83, 95% CI 0.59-1.18). Of note, the follow-up rate was 60.48% (967/1599) at that time point.

### Strengths and Limitations

Among the strengths of this study are its pragmatic design, with real-life conditions of clinical practice in terms of recruitment (inclusion criteria for patients and professionals), prescribed medication (patients were treated by their assigned practitioners at their usual consultations, without further restrictions), and minimum number of mandatory visits (baseline and at 6 months). Computer or internet literacy of patients was not checked at any point, and randomization was only conducted after their inclusion in the trial and collection of baseline data. The fact that professionals volunteered to participate can be detrimental to the outcomes because of a possible self-selection bias (participating workers may not be representative of the health staff due to a greater interest in tobacco addiction).

Usual practice was selected as the comparator due to being the standard treatment in our setting and because the chatbot attempts to reproduce the ideal face-to-face interaction between therapists and patients but as a novel technological support. This led to comparing 2 interventions of different intensities in terms of interaction time and number of contacts between the health worker or the chatbot and the patient. However, this comparison was justified by the pragmatic nature of the study.

The main outcome variable (continuous abstinence at 6 months) was biochemically validated, which increased the scientific accuracy of the results. So far, all clinical trials with apps [24,26,27] or chatbots [23] considered a patient to be abstinent based only on self-reports, with the exception of Pallejà-Millán et al [25].

The mentioned strengths reinforce the validity of the external outcomes, especially for health systems similar to the Spanish public health service. Although the Dejal@bot intervention cannot be directly delivered to the internet community without the intervention of a health professional to prescribe medication (if indicated), it could be provided by public or private health insurance systems. Alternatively, the pharmacological component of the intervention can be omitted to be able to implement it without the need for a health professional.

In terms of applicability, the system is ready for use and has enormous potential scalability, which could be improved with personalized technical assistance to facilitate accessibility, a key factor affecting the outcomes.

The main limitation of this trial was the dropout rate of 54.8% (281/513). Given the pragmatic design of the trial, no further midterm reinforcements or visits could be scheduled. Additionally, 38.8% (94/242) of the IG users never entered the chatbot. Losses to follow-up were homogeneous in both study arms, both quantitatively and in terms of participants’ characteristics after the intervention.

### Implications of the Study Findings/Implications of All Available Evidence

Dejal@bot showed its effectiveness in increasing nicotine abstinence rates in the long term compared with standard treatment provided by the usual doctor or nurse assigned to the patient, although these results must be interpreted with caution given the high dropout rate.

This intervention can facilitate patient access to high-quality treatments for the leading cause of preventable death (ie, tobacco smoking), saving costs for the health provider and reducing the workload for the professionals. At the time of this writing, with reduced mobility and social distancing due to the COVID-19 pandemic, this was especially appropriate and pertinent and could make a difference in the population’s health.

Further evidence is still required to assess the effectiveness of mHealth in smoking cessation. Although there are trials assessing the use of SMS text messages and apps for quitting, interventions using chatbots need to be evaluated, and qualitative studies about cost-effectiveness, usability, and satisfaction must be conducted. Additionally, determining the components that mainly affect effectiveness will be of interest to achieve behavioral changes and increased participation of users, because a strong association appears to exist between the time of use or accomplishment of tasks and dropout rates.

From the ethics perspective, the importance of high-quality studies evaluating these treatments must be highlighted, which will prevent the patient from being disfavored by incomplete, biased, or nonevidence-based interventions, and will also avoid decreased accessibility of certain population segments to quality therapies.

## Appendix

Multimedia Appendix 1 Differences between an app and a chatbot.

Multimedia Appendix 2 CONSORT-eHEALTH checklist (V 1.6.1).

Multimedia Appendix 3 Documento de información para el paciente.

Multimedia Appendix 4 Documento de Consentimiento Informado.

Multimedia Appendix 5 Screen capture.

Multimedia Appendix 6 TIDieR checklist.

Multimedia Appendix 7 Published articles about apps and chatbots to help in the tobacco cessation process.

## Abbreviations

AI: artificial intelligence
BOCF: baseline-observation-carried-forward
CG: control group
CONSORT: Consolidated Standards of Reporting Trials
ICT: information and communications technology
IG: intervention group
mHealth: mobile health
OR: odds ratio
ppm: parts per million
RR: relative risk
TIDieR: Template for Intervention Description and Replication
VAS: visual analog scale
