# Supplementary figures and images for: Effectiveness of a Conversational Chatbot (Dejal@bot) for the Adult Population to Quit Smoking: Pragmatic, Multicenter, Controlled, Randomized Clinical Trial in Primary Care
Source: JMIR Mhealth Uhealth. 2022 Jun 27;10(6):e34273. doi: 10.2196/34273 (PMC9274388; doi:10.2196/34273)

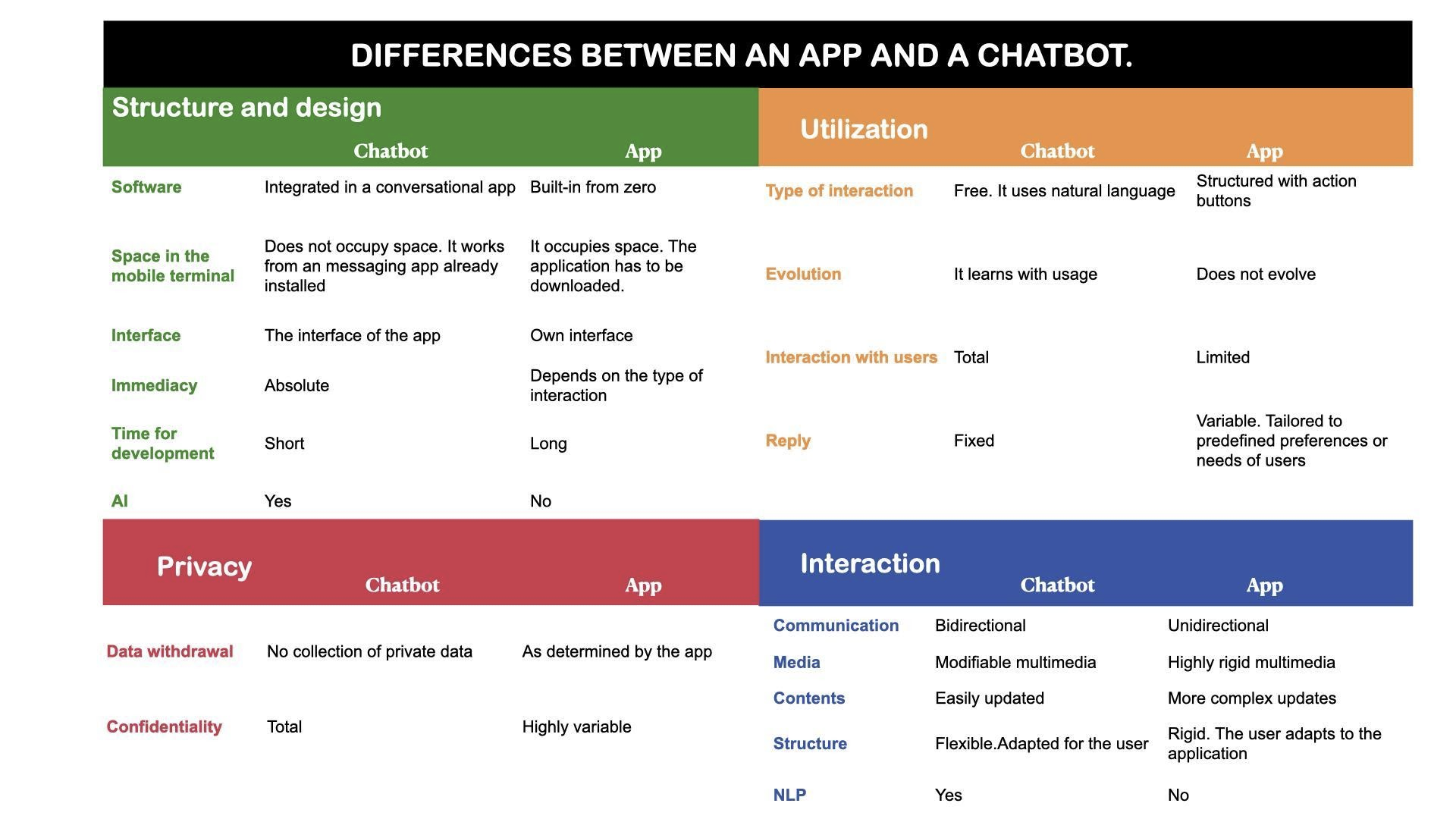

Supplement: Multimedia Appendix 1 [file mhealth_v10i6e34273_app1.png]

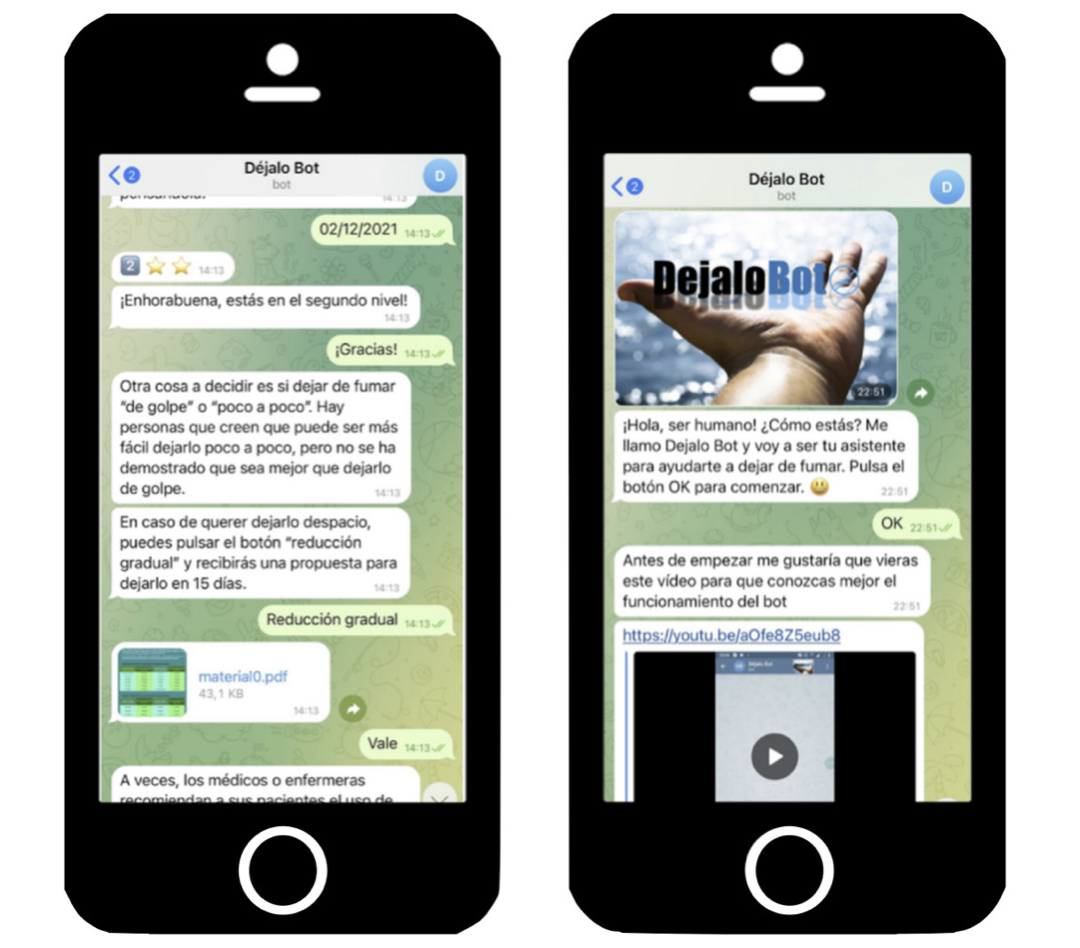

Supplement: Multimedia Appendix 5 [file mhealth_v10i6e34273_app5.png]
