# Supplementary material for: Effectiveness of a Conversational Chatbot (Dejal@bot) for the Adult Population to Quit Smoking: Pragmatic, Multicenter, Controlled, Randomized Clinical Trial in Primary Care
Source: JMIR Mhealth Uhealth. 2022 Jun 27;10(6):e34273. doi: 10.2196/34273 (PMC9274388; doi:10.2196/34273)
Supplement: Multimedia Appendix 3 [file mhealth_v10i6e34273_app3.docx]

## **Multimedia Appendix 3: Documento de información para el paciente**

| **TÍTULO DEL ESTUDIO** | “Efectividad de un bot conversacional para dejar de fumar en población adulta: ensayo clínico pragmático en Atención Primaria (Déjal@)”. |
| --- | --- |
| **CÓDIGO DEL ESTUDIO** | Expediente FIS-ISCIII: PI17/01942 |
| **PROMOTOR** | Fondo de Investigaciones Sanitarias (FIS). Instituto Carlos III. Gobierno de España. |
| **INVESTIGADOR** |  |
| **CENTRO** |  |

**1. Introducción.**

Nos dirigimos a usted para invitarle a participar en un proyecto de investigación que estamos realizando en varios centros de salud (los llamados “ambulatorios”) del sistema sanitario público de Madrid. El estudio ha sido aprobado por el Comité Ético del Hospital Doce de Octubre. La participación que le solicitamos es importante para obtener el conocimiento que necesitamos, pero antes de tomar una decisión debe:

Leer este documento entero.

Entender la información que contiene el documento.

Hacer todas las preguntas que considere necesarias a su médico o enfermera.

Tomar una decisión meditada.

Firmar el consentimiento informado, si finalmente desea participar.

Si decide participar se le entregará una copia de este documento y del consentimiento firmado. Por favor, consérvelos por si los necesitara en un futuro.

**2. ¿Por qué se le pide participar?**

Se solicita su colaboración por ser considerado usted un/una paciente fumador/a con disposición a aceptar ayuda por parte de su médico de familia o enfermera del centro de salud para dejar de fumar.

Su participación es voluntaria y puede decidir NO participar. Si decide participar, puede cambiar su decisión y retirar su consentimiento en cualquier momento, sin que por ello se altere lo más mínimo la relación con su médico o enfermera ni se produzca perjuicio alguno en su atención sanitaria.

**3. ¿Cuál es el objeto de este estudio? ¿En qué consiste?**

Su médico y/o enfermera está participando en un proyecto que estudia una forma diferente de abordar el problema del tabaquismo desde los centros de salud. El objetivo es evaluar cuan efectivo es un dispositivo llamado “bot conversacional” o “chat bot” diseñado de acuerdo a la evidencia científica para ayudar a las y los fumadoras/es a dejar de fumar comparándolo con la práctica clínica habitual en atención primaria (la ayuda prestada normalmente por su médico y/o enfermera).

Para ello se analizarán los datos de los primeros 460 pacientes que acepten participar, los cuales serán repartidos en dos grupos, llamados “de intervención” el uno y “de control” el otro. Las y los pacientes que resulten asignados al grupo “de intervención” podrán hacer uso del dispositivo a través de su teléfono móvil, y los que resulten asignados al grupo “de control” recibirán la ayuda que presten habitualmente su médico y/o enfermera.

Ni el médico o enfermera ni el/la paciente eligen el grupo que les corresponde, ya que se decide al azar por un ordenador. Esta distribución al azar es la manera de que los resultados obtenidos tengan alguna utilidad, es decir, que realmente sirvan para alcanzar los objetivos del estudio. Cada paciente tiene un 50% de posibilidades de ser asignado a cada uno de los dos grupos.

Independientemente del grupo al que usted sea asignado, su médico o enfermera pueden recomendarle un tratamiento farmacológico específico para ayudarle a dejar de fumar. Esto se hace siempre antes de la asignación al grupo del estudio, y depende de sus características como fumador/a y del criterio de su médico o enfermera.

**4. ¿Qué es un “chat bot” o “bot conversacional”?**

Es una aplicación informática dotada de inteligencia artificial que le permite interactuar con personas a través de una aplicación de mensajería instalada en el teléfono móvil, esto es, que puede responder a preguntas hechas por el usuario o puede hacer preguntas al usuario. En nuestro caso, ha sido diseñado por médicos expertos en tabaquismo con el fin específico de ayudar a las personas a dejar de fumar.

Usted lo que realmente verá, si es incluido en esa rama del estudio, es un chat de telegram (similar al whats app, pero con garantías de privacidad), al cual le podrá hacer preguntas sobre el proceso de abandono del tabaco y del cual recibirá información, recordatorios, enlaces a internet, vídeos, audios, y toda clase de material y consejos necesarios para dejar de fumar.

**5. ¿Qué tengo que hacer si decido participar?**

El estudio está dirigido a personas mayores de edad fumadoras que quieran dejar de fumar y que acepten ayuda para conseguirlo por parte de su médico o enfermera. También es necesario que tengan un teléfono móvil inteligente donde se pueda descargar la aplicación “Telegram” (gratuita) y que sepan usar aplicaciones de mensajería (tipo Whats app), por si les corresponde el grupo “intervención”

A todos los y las participantes se les realizará un mínimo de **DOS** visitas, la primera al inicio del proyecto y la segunda a los **6 meses**. Estas visitas serán realizadas por su médico de familia o enfermera del centro de salud. Las y los pacientes que estén en el grupo “control” recibirán además las visitas “extras” que estipulen con su médico o enfermera, según las dificultades del proceso. Los que estén en el grupo “intervención” recibirán información y consejos a través de un chat de Telegram en su teléfono móvil, por medio del cual pueden hacer las consultas que estimen oportunas.

Al inicio del estudio le harán una serie de preguntas sobre sus datos personales, su hábito de fumar y su salud y calidad de vida. La visita final es similar, añadiendo una cooximetría (prueba para detectar una substancia que se desprende del tabaco en el aire espirado, similar al test de alcoholemia que realiza la policía de tráfico). Esta segunda visita es muy importante en este proyecto por lo que, si ha decidido participar, le pedimos que si no pudiera acudir, nos lo comunique lo antes posible para tratar de conseguir otra cita en la fecha que mejor le convenga.

**6. ¿Qué riesgos o molestias supone su participación en el estudio?**

Le haremos dos entrevistas de una duración aproximada de unos 20 minutos en el mismo Centro de Salud donde usted es atendido habitualmente.

La cooximetría consiste en soplar el aire espirado en un tubo desechable, de forma similar a la prueba de alcoholemia que realiza la policía de tráfico. En muchos centros de salud y unidades de tabaquismo se realiza de forma habitual.

Los fármacos que su médico o enfermera pueden indicarle como ayuda para dejar de fumar son los habitualmente empleados, y no son objeto de este estudio.

**7. ¿Obtendré algún beneficio por mi participación?**

Participar en este estudio no le garantiza ningún beneficio, pero puede ayudarle a dejar de fumar. Además, usted puede ayudar a mejorar la atención a otros/as pacientes fumadores/as. Ni usted, ni los médicos/enfermeras, ni los/las investigadores/as del proyecto recibirán ninguna compensación económica por participar y desarrollar este estudio.

**8. ¿Cómo se van a gestionar mis datos personales?**

El equipo investigador se compromete al cumplimiento de la Ley Orgánica 15/1999, de 13 de diciembre de protección de datos de carácter personal y al Real Decreto que la desarrolla (RD 1720/2007). En la base de datos del estudio no se incluirán datos personales: nombre, número de historia clínica ni ningún otro dato que le pueda identificar. Se le asignará un código que sólo el equipo investigador podrá relacionar con su nombre. Sólo el equipo investigador tendrá acceso a los datos del estudio y nadie ajeno al centro podrá consultar su historial. Siempre se mantendrá la confidencialidad de sus datos de acuerdo a la legislación vigente.

De acuerdo a lo que establece la legislación de protección de datos, usted puede ejercer los derechos de acceso, modificación, oposición y cancelación de datos, para lo cual deberá dirigirse a su médico o enfermera. Si usted decide retirar el consentimiento para participar en este estudio, ningún dato nuevo será añadido a la base de datos, pero sí se utilizarán los que ya se hayan recogido con fines científicos. Las conclusiones del estudio se presentarán en congresos y a través de publicaciones científicas, pero se harán siempre con datos agrupados y nunca se divulgará nada que le pueda identificar como individuo.

La tecnología de bots en dispositivos móviles tiene varias ventajas frente a las aplicaciones (Apps) habituales: las Apps suelen basar su modelo de negocio en publicidad dirigida, lo que requiere acceso a datos personales (lista de contactos, aplicaciones instaladas o registro de llamadas). El bot no tiene acceso a ningún dato personal del usuario; de hecho no está instalado en el teléfono, sino que reside en la “nube” y el diálogo que el usuario tiene con él se produce mediante una App genérica de comunicación a la que se accede mediante un código personal.

A este respecto, “Telegram” (gratuita) ofrece las mayores garantías de seguridad por su encriptado y sus características técnicas (plataforma tecnológica estable, separación de datos, anonimización del usuario) y su modelo de negocio (opciones de pago). Además su uso no supone aprendizaje alguno.

**9. ¿Quién financia el estudio?**

Este proyecto se financia con fondos públicos a través de una beca de investigación procedente del Fondo de Investigaciones Sanitarias del Instituto de Salud Carlos III (ISCIII). Se trata del principal organismo público de investigación que financia, gestiona y ejecuta la investigación biomédica en España.

**10. ¿Se me informará de los resultados del estudio?**

Usted tiene derecho a conocer los resultados del presente estudio, tanto los resultados generales como los derivados de sus datos específicos. También tiene derecho a no conocer dichos resultados si así lo desea. Por este motivo, en el documento de consentimiento informado le preguntaremos qué opción prefiere.

**11. ¿Puedo cambiar de opinión?**

Tal y como se ha señalado, su participación es totalmente voluntaria. Puede decidir no participar o retirarse del estudio en cualquier momento sin tener que ofrecer explicaciones y sin que esto repercuta en su atención sanitaria. Basta con que manifieste su intención a su médico de familia o enfermera del centro de salud que le incluyó en el estudio.

**12. ¿Qué pasa si me surge alguna duda durante mi participación?**

En caso de duda o para cualquier consulta relacionada con su participación puede ponerse en contacto con el/la investigador/a responsable, Dr/Dra/Enfermera _______________ (incluir nombre y apellido del/la investigador/a), en el teléfono ___________ (indicar teléfono y horario) o por correo electrónico en la dirección _______________.

Muchas gracias por su atención. Si finalmente decide participar, le rogamos firme el Documento de Consentimiento Informado que se adjunta.

La descripción del estudio estará disponible en <https://clinicaltrials.gov>
