# Supplementary material for: Effectiveness of a Conversational Chatbot (Dejal@bot) for the Adult Population to Quit Smoking: Pragmatic, Multicenter, Controlled, Randomized Clinical Trial in Primary Care
Source: JMIR Mhealth Uhealth. 2022 Jun 27;10(6):e34273. doi: 10.2196/34273 (PMC9274388; doi:10.2196/34273)
Supplement: Multimedia Appendix 4 [file mhealth_v10i6e34273_fig.ocx]

**Multimedia Appendix 4: Documento de Consentimiento Informado.**

**Título del PROYECTO**: Efectividad de un bot conversacional para dejar de fumar en población adulta: ensayo clínico pragmático en Atención Primaria (Déjal@).

**Expediente FIS-ISCIII: PI17/01942**

Yo......................................................................................................................(nombre y apellidos del/la participante):

- He leído el documento de información que se me ha entregado.

- He podido hacer preguntas y he recibido suficiente información sobre el mismo.

- He hablado con .................................................(investigador).

- Comprendo que mi participación es voluntaria.

- Comprendo que puedo retirarme del estudio cuando quiera, sin tener que dar explicaciones y sin que esto repercuta en mis cuidados médicos.

- Presto libremente mi conformidad para participar en el estudio.

- Deseo ser informado sobre los resultados del estudio:

SÍ NO (marque lo que proceda).

- Soy consciente de que este consentimiento es revocable.

He recibido una copia firmada de este Documento de Consentimiento Informado.

| Firma del/la participante: |  |
| --- | --- |
| Fecha: |  |

Deseo que me comuniquen la información derivada de la investigación que pueda ser relevante para mí salud:

SÍ

NO

He explicado la naturaleza y el propósito del estudio al paciente.

| Firma del investigador/a: |  |
| --- | --- |
| Fecha: |  |
