# Supplementary material for: Effectiveness of a Conversational Chatbot (Dejal@bot) for the Adult Population to Quit Smoking: Pragmatic, Multicenter, Controlled, Randomized Clinical Trial in Primary Care
Source: JMIR Mhealth Uhealth. 2022 Jun 27;10(6):e34273. doi: 10.2196/34273 (PMC9274388; doi:10.2196/34273)
Supplement: Multimedia Appendix 6 [file mhealth_v10i6e34273_app6.docx]

**Multimedia Appendix 6: TIDieR checklist.**

|  | **BRIEF NAME** |
| --- | --- |
| 1. | Helping the adult smoking population quit via a conversational chatbot in the smartphone. |
|  | **WHY** |
| 2. | The intervention strategy was based on the 5A’s of the US clinical practice guideline (2), which combine behavioral and pharmacological treatments throughout several follow-up visits, whether via a chatbot or in-person at the consultation of the assigned health worker. |
|  | **WHAT** |
| 3. | Materials: No physical or informational materials were used during the intervention or the training of professionals who delivered the intervention.  Materials with the information provided to patients can be found in Multimedia Appendix 1. |
| 4. | Procedures: During the recruitment phase, all patients that met the inclusion criteria were interviewed in person about their tobacco consumption and received advice about smoking cessation from their doctor or nurse, who also evaluated their willingness to quit. Those who accepted to attempt cessation during the following month and take part in the trial were randomly allocated into the intervention or control group.  Patients assigned to the control group were offered usual clinical practice to help in the cessation process, which consists of a minimum of one visit before the cessation date and another visit after one month. Opportunistic controls or at the criterion of the professional could also be scheduled, with different duration depending on the professional or patient needs.  Patients in the intervention group were offered an intervention with similar content to the control group but delivered via a chatbot in the smartphone.  Both groups received an intervention including: advice to quit smoking based on evidence-backed techniques of different kinds (cognitive-behavioral, motivational, relapse preventing, and problem-solving); information about prescribed medication for quitting; and coping strategies for problems during the cessation process.  No enabling or support activities were conducted. |
|  | **WHO PROVIDED** |
| 5. | No specific training was delivered to the included doctors and nurses. Given the fact that they volunteered to participate, it is likely that their expertise level and interest in tobacco addiction are superior to the average health worker. |
|  | **HOW** |
| 6. | The intervention was delivered individually, whether via the smartphone or face-to-face with their assigned health professional. |
|  | **WHERE** |
| 7. | The recruitment, basal visit, and final visits took place at the healthcare center consultation of the health worker. Patients in the intervention group decided where they connected to the chatbot. |
|  | **WHEN and HOW MUCH** |
| **8.** | The contact frequency established by the bot varied depending on the cessation date and patient characteristics (personal choice, type of consumption, risk situations, prescribed drug, abstinence symptoms, and evolution with abstinence). The patient could contact the bot at any moment or place and decide the intensity, schedule, and frequency of contacts.  In the control group, a minimum of one visit before the cessation date and one visit after one month were scheduled , plus opportunistic controls or at the criterion of the professional, with different duration depending on the health worker or patient needs. |
|  | **TAILORING** |
| **9.** | The intervention was not designed to be personalized, titrated, or adapted, circumstances that were determined depending on the criterion of patients in the intervention group or professionals in the control group. |
|  | **MODIFICATIONS** |
| **10.^ǂ^** | The intervention did not undergo modifications during the course of the study. |
|  | **HOW WELL** |
| **11.** | Adherence to the intervention was assessed via the number of contacts, duration of contacts, and number of patients who completed the trial. These variables were automatically measured by the chatbot or the data collection notebook.  No strategies were implemented to maintain or improve fidelity. |
| **12.^ǂ^** | Intervention adherence was not assessed, but the intervention was delivered as planned.  Dropout rate was higher than expected, but, nevertheless, similar to other studies and to CG. In fact, many patients do not attend face-to-face visits despite an initial commitment to quit smoking. |
