# Supplementary material for: Effectiveness of a Conversational Chatbot (Dejal@bot) for the Adult Population to Quit Smoking: Pragmatic, Multicenter, Controlled, Randomized Clinical Trial in Primary Care
Source: JMIR Mhealth Uhealth. 2022 Jun 27;10(6):e34273. doi: 10.2196/34273 (PMC9274388; doi:10.2196/34273)
Supplement: Multimedia Appendix 7 [file mhealth_v10i6e34273_app7.pdf]

Multimedia Appendix 7. Published articles about apps and chatbots to help in the tobacco cessation process.

Name	Apple or Google stores	Company/Institution	Clinical evidence	Reported results
SmartQuit	Yes	Fred Hutchinson Cancer Research Center (USA)	NCT03174730 (N=398) NCT02724462 (N=2503)	Acceptance and commitment therapy. Cognitive and behavioral therapy. SmartQuit 2.0 yielded an overall quit rate of 21% and a reduction rate in cigarette consumption of 75%.
SmokeFree28	Yes	Cancer Research UK Health Behaviour Research Centre (UK)	Pilot test (N=1170)	Behavior-change techniques 18.9% were recorded as abstinent from smoking for >28 days. Further evaluation by means of a randomized trial appears to be warranted.
SmokeFree and SmokeFree + Chatbot	Yes	David Crane	Pilot test (N=6111)	Participants offered the addition of the supportive chatbot (15.8%) had 2.44 times greater odds of being abstinent at the one-month follow-up survey compared with the standard version of the Smoke Free app (7.1%).<br>ChatBot feature is still under development or dismissed.
Quit Genius	Yes	Quit Genius (USA)	Pilot test (N=4144) Validated in 6 peer-reviewed studies.	Cognitive behavioral therapy. It has helped 30k people to cease smoking Respondents had completed an average of 60% of the program in the Quit Genius app. Of the respondents, 36.3% reported having quitted smoking successfully after using the Quit Genius app.
QuitBot	No (Facebook Messenger chatbot)	Fred Hutchinson Cancer Research Center (USA)	NCT03585231 (N= 415) NCT04308759 (N=1520)	New enrollments are currently closed for the first QuitBot Study. The second trial is expected to start in April 2021 and will compare the QuitBot with the Texting Smoking Cessation Program.
Quitly	No (Facebook Messenger chatbot)	Cancer Institute NSW (New South Wales) (Australia)	No	Does not apply. It is worth mentioning that Quitly is a free-to-use chatbot.
